# Value-added fabrication of NiO-doped CuO nanoflakes from waste flexible printed circuit board for advanced photocatalytic application

**DOI:** 10.1038/s41598-022-16614-4

**Published:** 2022-07-16

**Authors:** Rumana Hossain, Rasoul Khayyam Nekouei, Abdullah Al Mahmood, Veena Sahajwalla

**Affiliations:** Centre for Sustainable Materials Research and Technology, SMaRT@UNSW, School of Materials Science and Engineering, UNSW Sydney, Sydney, Australia

**Keywords:** Pollution remediation, Materials for energy and catalysis

## Abstract

The disposal of electronic waste (e-waste) presents a number of environmental problems. However, there are great opportunities to use this problem waste as a source of value-added metals. These metals could be recovered and transformed for use in beneficial applications, such as the manufacture of nanomaterials for the generation of hydrogen through thermodynamic water-splitting. This study used microrecycling techniques to synthesise Nitrogen oxide (NiO) doped copper oxide (CuO) nanoflakes from waste flexible printed circuit boards (FPCBs) using microrecycling techniques. Several precise characterisation and experimental analysis were used to validate the synthesised nanoflakes’ phase purity, surface chemistry, morphology and optical properties. XRD analysis confirmed the nanoflakes produced in the system were predominantly Tenorite, CuO (98.5% ± 4.5) with a dopant of NiO (1.5% ± 0.1). The nanoflakes had a specific surface area of 115.703 m^2^/g and mesoporous structure with an average pore diameter of 11 nm. HRTEM analysis confirmed that the nanoflakes were not a single structure but assembled from 2D nanorods. The width of the nanorods varied from ∼ 10 to 50 nm, and the length from ∼ 30 to 80 nm. After rapid thermal processing, the photocurrent response of the synthesised material was assessed, revealing a higher photocurrent density (− 1.9 mA/cm^2^ at 0.6 V vs. reversible hydrogen electrode (RHE) under 1.5G AM). Mott Schottky analysis and electrochemical impedance spectroscopy showed that the synthesised nanomaterial had the potential thermodynamic water-splitting capability. These results were an encouraging indication of the promise of techniques which use e-waste to produce nanomaterials with valuable properties. This has the potential to both decrease problem waste and preserves dwindling natural resources.

## Introduction

As natural resources decline, global industry and manufacturing need to embrace novel strategies which replace conventional resources with materials that are transformed from wastes. One of the most problematic waste streams is electronic waste (e-waste). This comprises many valuable metals and non-metals. For example, flexible printed circuit boards (FPCBs) contain more than 99% pure copper embedded in non-metals such as polyimide/polyamide and resin^1^, in which a Ni-based emulsion is widely used as a surface finish for FPCBs, particularly in soldering locations to protect from oxidation during soldering. FPCBs typically are made as a large sheet. During the final production stage, they are punched and cut to the desired shape and size, leaving behind a large amount of waste rich in valuable Cu metal. This Cu could be recovered by a thermal disengagement technique (TDT) and be further used in other industrial applications^2,3^. In this study, we have used Cu recovered from waste FPCBs to synthesise CuO nanomaterial and evaluated the performance of the synthesised material thermodynamic water splitting.

CuO and Cu_2_O as transition metal oxides are two semiconducting phases of copper oxide^4^. The direct bandgap for Cu_2_O is 2.1 eV^5^, and it is used in a number of applications, including photovoltaics^6^, supercapacitors^7^, photocatalysis^8^, and sensors^9^. CuO, on the other hand, is favoured for photoelectrochemical (PEC) applications as a result of its excellent light absorption capability and high physical stability. CuO has prospective applications in numerous fields, including water-splitting photoelectrochemical processes^3,4^, photovoltaics^5,6^, supercapacitors^7,8^, photocatalysis^9,10^, photodetectors^11–13^, batteries^14,15^, and biosensors and chemicals^11,16,17^. The bandgap of CuO can be engineered from 1.2 to 1.7 eV^10^. This allows the semiconductor to absorb the solar spectrum over a wider range of wavelengths, making it an attractive nominee for photovoltaic applications. Minor carriers excited by the photoactivity are guided to the interface of the semiconductor and electrolyte in PEC water splitting, where they undergo a redox reaction and generate hydrogen from the holes of n-type semiconductors or oxygen from electrons from p-type semiconductors^11^. In water-splitting, n-type materials such as ZnO^12^, Fe_2_O_3_^13^, and TiO_2_^14^ operate as oxygen-evolution electrodes, and p-type substances such as CuBi_2_O_4_^15^, InP^16^, and WSe_2_^17^ act as hydrogen-evolution electrodes. CuO is a strong photocathode for hydrogen generation because of its p-type conductivity and precise conduction band location^18^.

Various researchers have proposed different methods for producing ultra-fine Cu powders using PCB waste materials^19–21^. The proposed methods include electrokinetic processes^22^, chemical reduction^23^, electrochemical processes^24^, and cementation^25^.

To synthesise the NiO doped CuO, researchers synthesised NiO by heat-treating the basic nickel carbonate. The pure NiO samples were used with the copper nitrate and performed chemothermal process to have the final product^26,27^. In another study, researchers used analytical grade Cu(NO_3_)·3H_2_O and Ni(NO_3_)·6H_2_O and performed stepwise calcination to produce CuO-NiO catalyst^28^. Ni-doped CuO nanoparticles were produced by adding NiSO_4_·6H_2_O to CuSO_4_·5H_2_O solution and by utilising chemothermal reduction^29,30^ or microwave irradiation^31^. In the literature, Cu was leached in the initial step, and nanomaterials were prepared in the second step. In this research, we report the production of NiO-doped CuO nanoflakes (without Cu_2_O) from waste FPCBs using a microrecycling process consisting of a combined chemical route followed by a simplified thermal route (i.e., TDT^1,2^). We evaluated the suitability of the synthesised nanoflakes as light-absorbing materials for energy-harvesting applications.

In this study, the bandgap energy for CuO was measured as 1.57 eV, which is higher than the value for bulk CuO (1.2 eV)^32,33^ and lower than NiO nanoparticles (3.8 eV)^34,35^. The shift of the energy from one level to another can be attributed to quantum confinement in the nanocrystal structure. It has also been reported by several researchers that the combination of NiO and CuO can shift the bandgap to a higher region compared to the pure CuO^36,37^. The theoretical bandgap for an ideal material for water splitting is reported to be ~ 2 eV^38^. In the real field application, this range varies from 1.23 to 3 eV^39^. The actual bandgap of the bulk CuO is 1.2 eV has been considered as low and the bandgap of NiO is 3.8 eV has been considered as high for water splitting applications. The synthesised CuO nanoflakes exhibited a bandgap of 1.57 eV, making them a good candidate for the energy harvesting application.

## Experimental

### Fabrication of CuO nanoflakes

A thermo-chemical route was adopted to synthesise NiO-doped CuO nanoflakes, as represented in Error! Reference source not found.. In the first step (thermal process), the waste polymer-laminated thin Cu sheet was converted into the disengaged elements of solid Cu and thermally-degraded carbonaceous compounds from the laminated polymers. Thermal disengagement technology was used to process FPCBs. In this technique, a controlled (typically inert) environment was created by a continuous supply of argon gas. The polymers are prone to oxidation and emit carbon into the atmosphere if they come into contact with oxygen. In this technique, there is an artificial deficiency of oxygen, so the polymers devolatilised and degraded into solid carbon deposited on the surface of the Cu. Therefore, in this thermal process, the two main elements of Cu and carbon can be recovered.

The second step (chemical route), involved performing a series of chemical treatments on the thermally-degraded elements generated from the first step. The mixture of Cu and carbon was dissolved in an acid solution (a mixture of sulfuric and nitric acid), while carbon particles remained unreacted and floated on top of the solution. The carbon particles were removed from the surface of the solution. The overall reaction by which the Cu was digested in the acidic media is shown below:

1 $$ {\text{3Cu }} + {\text{3H}}_{{2}} {\text{SO}}_{{4}} + {\text{ 2HNO}}_{{3}} \to {\text{ 3CuSO}}_{{4}} + {\text{ 2 NO }} + {\text{ 4 H}}_{{2}} {\text{O}} $$

**Figure 1:**
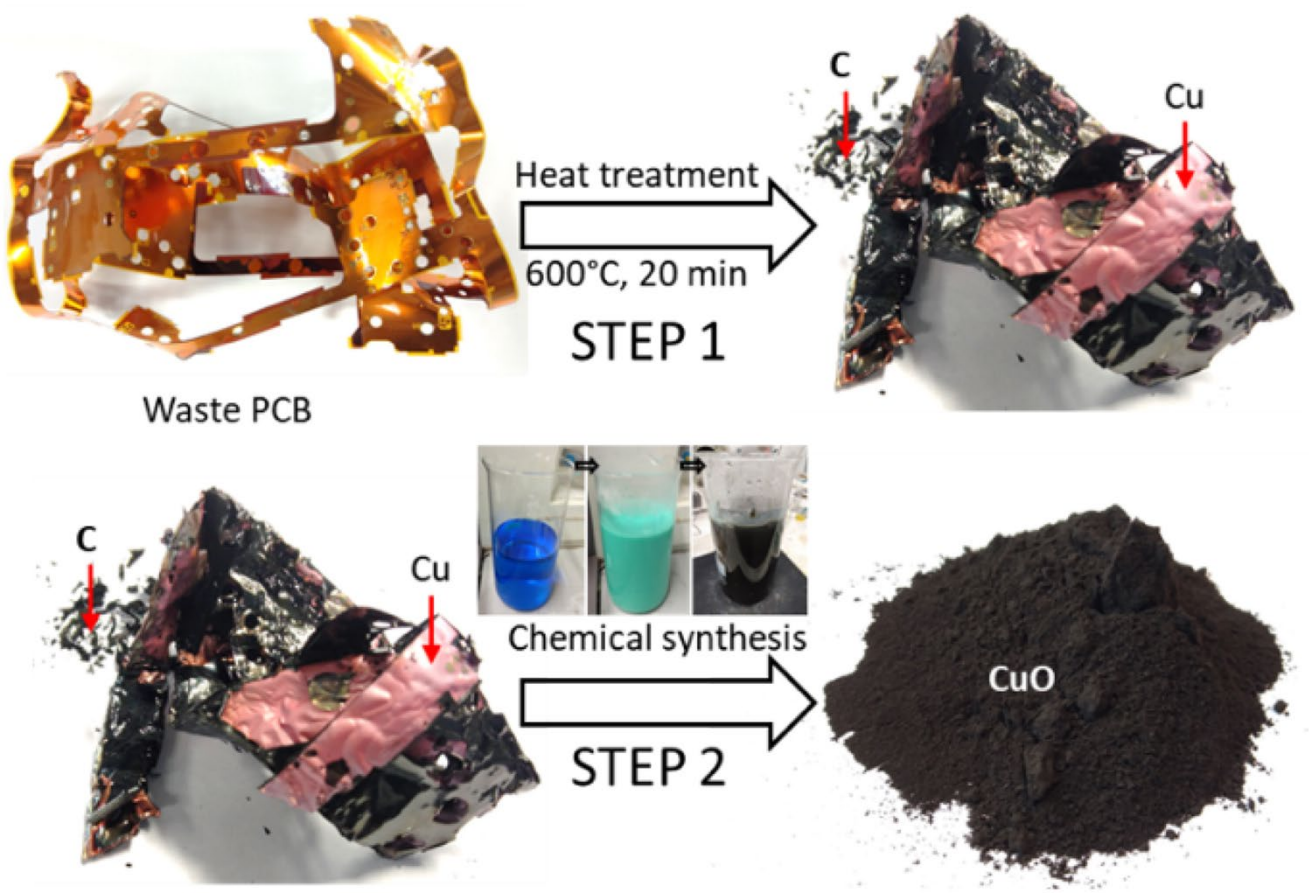
Steps for the synthesis of NiO-doped CuO nanoflakes.

The blue solution in Fig. 1 (Step 2) mainly consists of CuSO_4._ A NaOH solution was slowly added to the blue solution. While adding NaOH, the colour of the solution first changed to aqua. When almost all the NaOH had been added to the solution the colour changed to black, and a visible CuO residue was observed. A series of filtrations were carried out to clean the CuO and following filtration, it was heated at 80 °C to remove moisture. The pH value of the solution was 8.

2 $$ {\text{CuSO}}_{{4}} + {\text{ 2NaOH}} \to {\text{Cu }}\left( {{\text{OH}}} \right)_{{2}} + {\text{ Na}}_{{2}} {\text{SO}}_{{4}} $$

3 $$ {\text{Cu }}\left( {{\text{OH}}} \right)_{{2}} + {\text{ Heat}} \to {\text{CuO }} + {\text{H}}_{{2}} {\text{O}} $$

### Electrode fabrication

Film electrodes made of CuO nanoflakes (for energy harvesting applications) were deposited via drop-casting (using a micropipette) of 20 μl of conductive ink on a fluorine-doped tin oxide (FTO) surface-coated glass with exposed surface area (~ 1 cm^2^). Before the deposition, the FTO substrate was thoroughly rinsed and cleaned with distilled water, acetone, and ethanol in a sonicating system with a water bath. To make the conductive ink, 600 µl of isopropanol and 600 µl of Deionised water were added sequentially to 20 mg of activated material. The tube containing this suspension was sealed and magnetically stirred for 0.5 h, followed by sonication for 1 h. The scheme is provided in the supplementary to demonstrate the electrode fabrication (Figure S1).

### Characterisation of photoelectrode material

The elemental composition of the nanoflakes was characterised by inductively-coupled, plasma-based optical emission spectroscopy (ICP-OES), using a Perkin Elmer OPTIMA 7300 after digestion in nitric acid. The composition of the nanoflakes was also confirmed with X-Ray Photoelectron Spectroscopy (XPS) analysis, which was carried out in a Thermo Scientific ESCALAB250Xi machine, using an Al K_α_ X-ray source. X-ray diffraction (XRD) with CoK radiation was used to analyse the phase detail and crystallinity of the product. The microstructural characterisation of the synthesised NiO doped CuO nanoparticles was carried out with a field emission scanning electron microscope (FE-SEM)—FEI Nova NanoSEM 450 FE-SEM along with energy dispersive spectroscopy (EDS). Microscopic analysis was carried out using high-resolution transmission electron microscopy (HR-TEM) and selected area electron diffraction (SAED) patterns. A Lambda 950 spectrometer (PerkinElmer) was used to collect the diffuse spectra (UV–Vis) in the 300 to 1000 nm range. The surface area of the nanoflakes was measured using Brunauer–Emmett–Teller (BET) N_2_ adsorption/desorption technique at 77.4°K and the pore structure was measured using the Barrett-Joyner-Halenda (BJH) method. UV–Vis spectroscopy of CuO was carried out with a double-beam Lambda 1050 UV–Vis–NIR spectrometer (PerkinElmer, WB InGaAs, UK). The instrument used for the nanosamples with variable thickness was an integrating sphere (150 mm), which was used to collect the diffused, transmitted, and reflected scattering in the wavelengths from 250 to 2000 nm and 5.0–0.6 eV. Before performing UV–Vis analysis of the actual sample, a standard sample was analysed, and the instrument was calibrated to ensure the accuracy of the measurement. A Raman spectrometer (REINSHAW, inVia) was used to measure the photoluminescence characteristics of the CuO-based material. The spectrometer was connected to a microscope, known as a Raman microscope. Using the microscope, the laser was focused onto the sample's surface and the scattered light from the sample was collected by the Raman spectrometer. The wavelength of the laser for the photoluminescence analysis was 325 nm with a grating of 1800 I mm^−1^ (Vis).

### Photoelectrochemical analysis

A three-electrode cell adjustment for the potentiostat/galvanostat (VSP-300, BioLogic, USA) workstation was used to characterise the electrochemical behaviour of the synthesised sample at room temperature. The working electrode for this system was an FTO-supported film, the counter electrode was a platinum spiral wire, and the reference electrode was a typical calomel electrode (known as a standard calomel electrode, SCE). In the illuminated and dark atmosphere, cyclic volumetry (CV), linear volumetry (LV), and electrochemical impedance spectroscopy (EIS) were carried out. Detailed parameters are outlined in the corresponding sections of this study. The system configuration for the EIS was 10 mV, between the frequencies of 100 mHz and 100 kHz, with an open circuit potential (OCP) after leaving the sample in the solution for 20 min to reach a constant OCP. The working electrode (thin film on FTO) was directly illuminated using a G2V pico LED solar simulator under 1.5G air mass (AM), 1 Sun equal to 100 mW cm^−2^, with wavelengths only between 400 and 800 nm irradiating the sample. The photoactivity of the film was measured in dark conditions and under manually-chopped and full illumination. In photocatalytic applications, a 2 M KOH solution was used as the electrolyte. The following equation was used to transform the potential of the SCE to that of a reversible hydrogen electrode (RHE):

4 $$ {\text{RHE}}\;({\text{V}}) \, = {\text{ SCE}}\;{\text{(V) }} + \, 0.{24}\;{\text{V}}\;vs. \, \;{\text{RHE }} + \, 0.0{59 } \times {\text{ pH }}\left( {{\text{which }}\;{\text{was}}\;{ 14}.{3}\;{\text{ in}}\;{\text{ this }}\;{\text{research}}} \right) $$

## Results and discussion

### Crystallographic structure, morphological, and spectroscopic analysis of NiO-doped CuO nanoflakes

**Figure 2:**
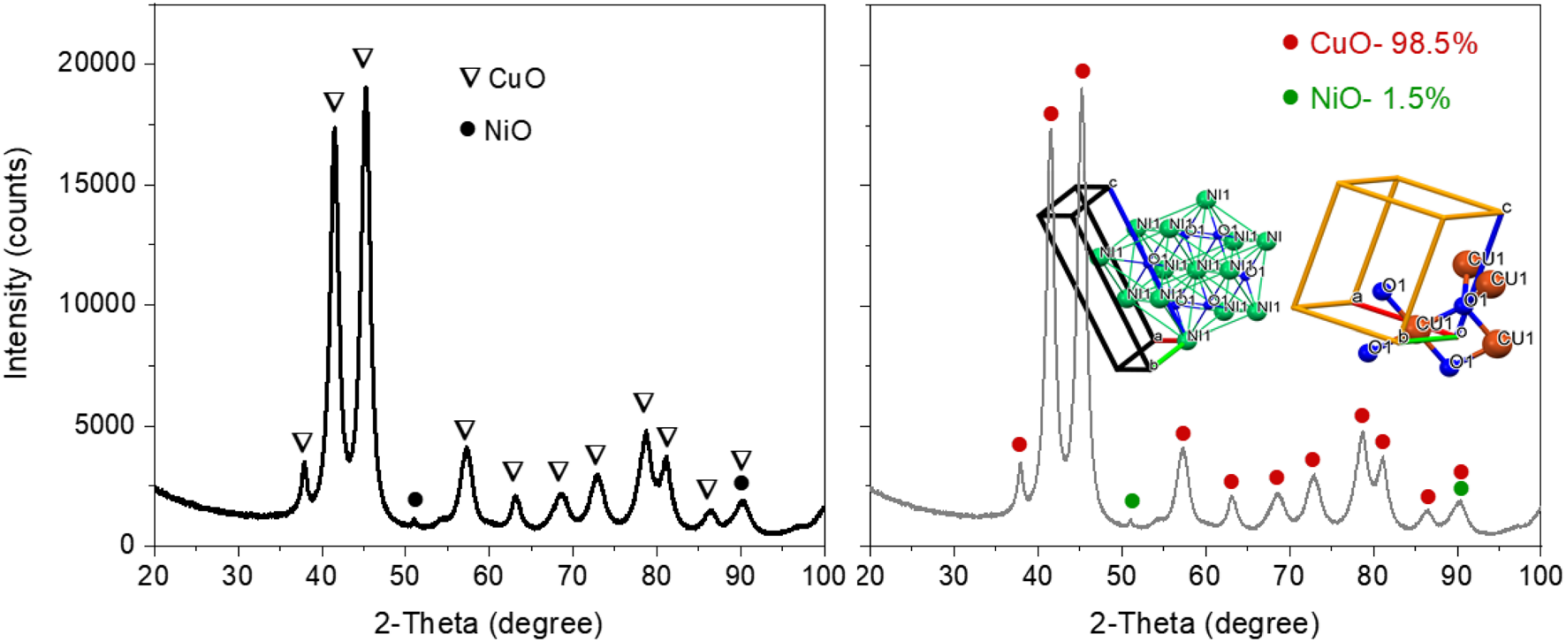
(**a**) XRD pattern of nanoflakes with all peaks explained by CuO as a major phase and NiO as a trace phase. (**b**) Rietveld fit analysis for composition with relative abundances of Tenorite (CuO) and NiO. Inset: The unit cell of CuO.

The recorded XRD pattern of CuO-based nanomaterial was processed using HighScore plus software (supplier: PANalytical BV) for a candidate search match in the ICSD database, followed by a Rietveld fit to identify and quantitatively establish the abundances of Tenorite (CuO) and NiO (as shown Fig. 2). An acceptable Rietveld fit was obtained with these two phases (Rwp: 9.91). Nanoflakes produced in the system were confirmed to be predominantly Tenorite, CuO (98.5% ± 4.5) with traces of nickel oxide, NiO (1.5% ± 0.1).

After refinement, the lattice parameters were calculated to be: a = 4.696(2) Å, b = 3.414(3) Å, c = 5.137(9) Å, and β = 99.2°, with cell volume 81.31 Å^3^. This can be compared to ICSD data card values of a = 4.683(0) Å, b = 3.459(0) Å, c = 5.130(0) Å, and β = 99.309° with cell volume 81.29 Å^3^ for CuO. A full pattern Rietveld fit with size^31^ and strain-specific parameters^32^ was applied to approximate the average size of the crystallites and developed microstrain. The instrument broadening was taken into account by measuring a Silicon standard sample (Si 640c). The CuO crystallite size was found to be 7.7 nm, and the associated microstrain was estimated as 0.207%. The following equation was used to calculate dislocation density (δ), which is defined as the length of a dislocation line per unit metre square of the crystal:

5 $$ \delta = \frac{1}{{D^{2} }} $$

The obtained value of dislocation density, dislocation length, and lines per unit volume of a crystal structure is: δ = 0.0169 and strain ɛ_str_ = 0.207%. This signifies a superior crystallisation and good quality of the CuO nanoflakes, which may be appropriate for applications in photovoltaics. This study found that the measured strain resulting from the dislocation density and lattice dislocation was deficient and had no effect on the broadening peaks for CuO tetrapods.

**Figure 3:**
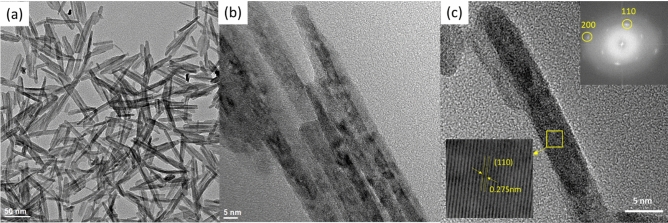
(**a,b**) Bright-field TEM image, (**c**) lattice structure of nano CuO flakes with electron diffraction pattern.

The microstructure, crystal structure and elemental mapping of the NiO-doped CuO nanoflakes were investigated using HR-TEM imaging, SAED, and TEM-EDS analysis represented in Fig. 3. The nanoflakes demonstrate an irregular pattern and feather-like morphology with a varied flake size. The irregular morphologies of the flakes are formed by assembling many 1D nanorods visible in the HR-TEM images. As the flakes are not a single structure but the assembly of several 1D nanorods, the width varies from ∼ 10 to 50 nm and the length varies from ∼ 30 to 80 nm. From the HR-TEM images, it is clearly understood that many rod-like particles are accumulated together to develop the structure of the nanoflakes which could be ascribed to the well-known Ostwald ripening phenomena^40^. In this procedure, at the beginning of the synthesis of the nanoflakes, particles ranging from small to large are generated in the non-equilibrium solution. The smaller particles dissolve easily and create free atoms which are transferred to the surface of the bigger particles. This process continues because the bigger crystals are energetically favourable compared to the smaller crystals and promote more solubility for the smaller crystals. The reprecipitation of the smaller crystal on the surface of the larger crystals creates a compact structure which is favourable for the use of this CuO in solar cells for the transportation of photocurrent. The CuO nanoflakes show well-defined fringes, which are attributed to the single crystal of CuO. The measured lattice spacing was 2.75 Å, which is attributed to (110) interplanar spacing. The SAED pattern also confirms the absolute monoclinic structure, which corresponds to the XRD pattern. The TEM-EDS mapping shows Cu and O distribution in the nanoflakes.

**Figure 4:**
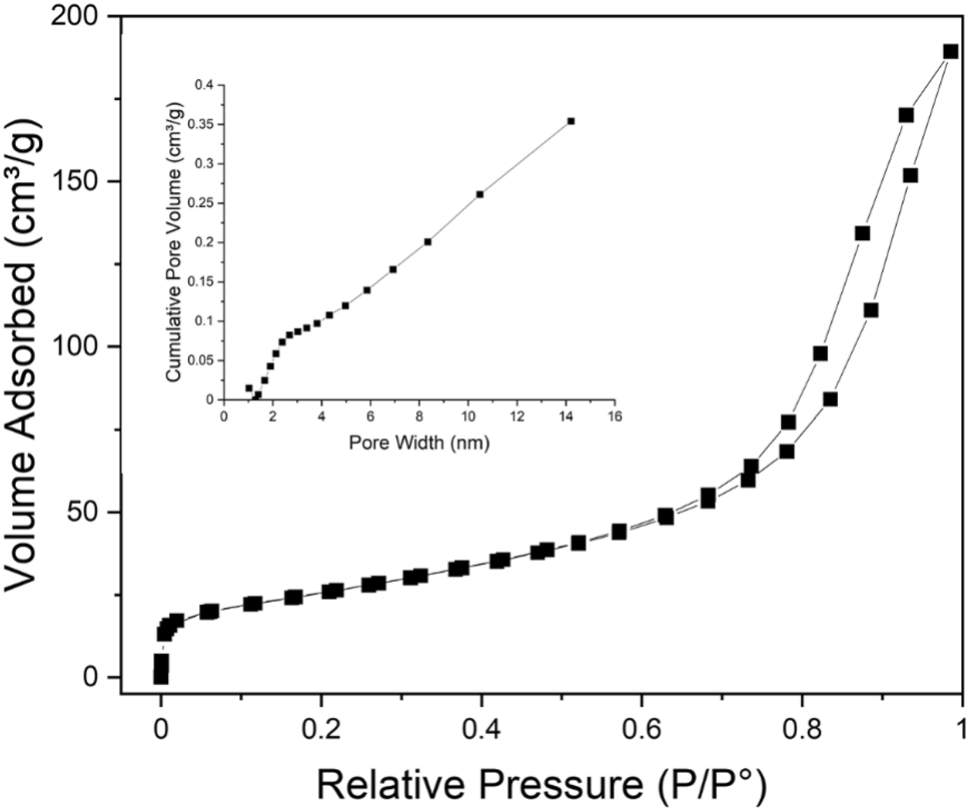
Isotherms for N_2_ adsorption–desorption at 77 °K and pore size distribution (BJH) of the synthesised nano CuO.

The porosity and the specific surface area of CuO nanoflakes were measured using N_2_ isotherms for adsorption–desorption at 77.4°K and pore size distribution (BJH) measurements. The isotherm exhibits type IV hysteresis, as represented in Fig. 4, and the relative pressure (P/P_o_) and loop are from 0.65 to 1.0, which further indicates the structure is mesoporous. The collected CuO nanoflakes have a specific surface area of 115.703 m^2^ g^−1^, and the pore size distribution (BJH) indicates that the CuO nanoflakes have a mesoporous structure with an average 6 nm pore diameter.

**Figure 5:**
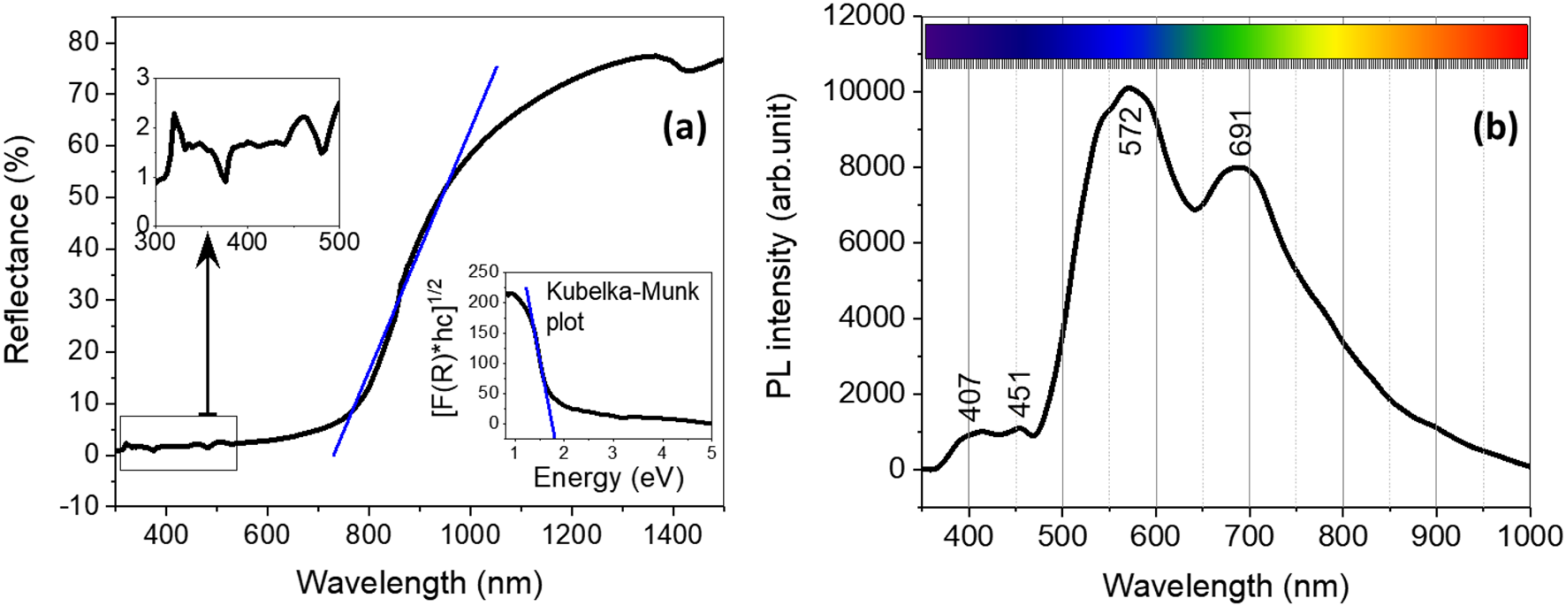
(**a**) UV–Vis spectrum (inset: Kubelka–Munk plot) and (**b**) Photoluminescence analysis for CuO.

The surface chemistry of the CuO nanoflakes was explored using UV–Vis spectroscopy, represented in Fig. 5a. The reflectance characteristics at different wavelengths were also analysed with UV–Vis spectroscopy. Due to the initial stability and lance changing for UV–Vis spectroscopy setup, there are a few smaller humps enlarged with scales. Those humps are very common in UV–Vis spectroscopy for nanomaterials in the range of 300–500 nm. The nanoflakes show around 70–75% reflectivity in the visible and infrared region, which is a good indication of the applicability of this material in solar energy harvesting^41^. The indirect bandgap can be estimated using a Kubelka–Munk plot or Tauc plot (shown in the inset of Fig. 5a). The relationship between the reflectance and estimated bandgap can be written as:

6 $$ F\left( R \right) = \frac{{\left( {1 - R} \right)^{2} }}{2R} $$

where R is the reflectance percentage of CuO measured by the UV–Vis spectroscopy at different wavelengths. The bandgap measured for this CuO by the Kubelka–Munk plot is ~ 1.57 eV, which is relatively higher than that of the bandgap of bulk CuO^42^. The increase in the energy of the bandgap can be ascribed to quantum confinement in the nanocrystalline arrangement. This quantum confinement can be caused by the presence of another oxide (for example, NiO) in the original composition of CuO^36,37^.

The photoluminescence of a material varies with factors including the composition, synthesis technique, and storing system. The spectra can be derived from the combination of the free transporters in the defect in the energy state. It is a sign to estimate the bandgap of the material. The photoluminescence spectra for CuO are shown in Fig. 5b. There are few emission peaks at varying wavelengths, and all four major peaks are in the visible range. The peak at 407 nm is for violet emission, at 451 nm for the blue region, and the significant one at 572 nm can be attributed to the green emission region^43^. Some other minor peaks are derived from the free excitation of electron–hole pairs and their recombination^42^. The photoresponse of NiO is also identified at 691 nm. This emission peak is considered to be due to the participation of NiO in the mixture of CuO nanoflakes^42,44^.

### Electrochemical properties of CuO nanoflakes

**Figure 6:**
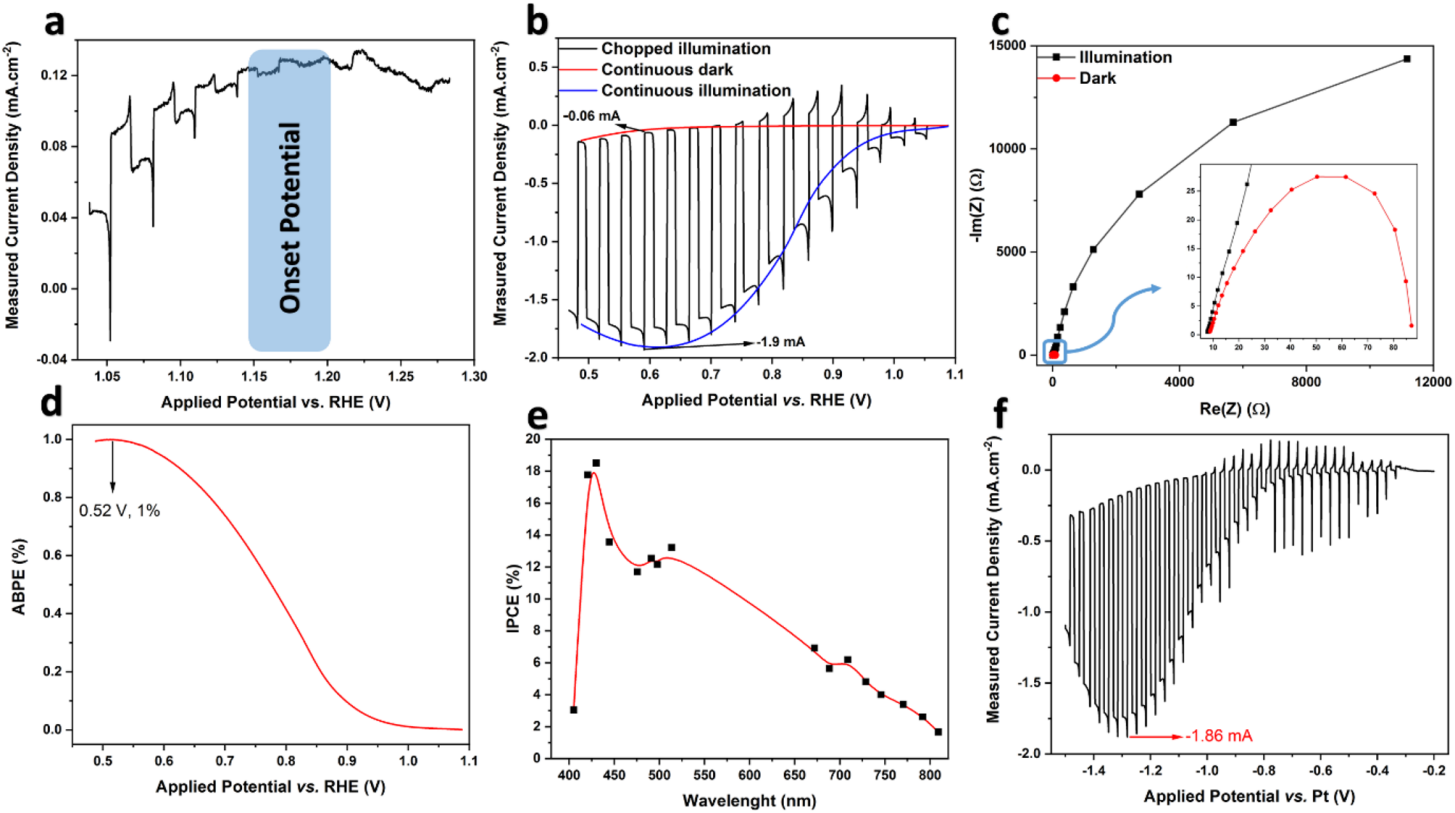
Photoelectrochemical results of the film electrode made from heat-treated (at 400 °C for 5 min) NiO-doped CuO nanoflakes employed in 2 M KOH solution and under illumination intensity of AM 1.5 G; (**a**) photocurrent (j–V) plot of the film at low current density for identification of onset potential, (**b**) three-electrode photocurrent plot of the film, the scanning rate of 20 mV s^−1^ and chopped light illumination at ~ 0.5 Hz frequency, (**c**) Nyquist plot of the film at − 0.3 V versus SCE under illumination and in dark conditions, in the frequency range of 0.1 Hz to 0.1 MHz under 10 mV AC amplitude, (**d**) calculated functional bias photon to electron conversion efficiency (ABPE) of the film electrode extracted from the data obtained from continuous illumination and continuous dark conditions [see (**b**)], (**e**) photon incident to current conversion effectiveness (IPCE) for the film electrode at − 0.3 V versus SCE, and (**f**) two-electrode photocurrent plot of the film.

In the next step, the nanoflakes were rapidly heat-treated at a low temperature (5 min at 400 °C). They were then used to make ink for the fabrication of a film electrode on the surface of a current collector (FTO). In the first step, the photocurrent onset potential (E_onset_) and the capability of the film to generate photocurrent were examined. E_onset_ is the potential where the minority electron carriers in the photocathode trigger a Faradic reaction (which in this research is hydrogen evolution reaction (HER)) at the interface of solid/liquid^45^). Figure 6a illustrates the j–V plots of the film at a very low current density, where the small photocurrents can be identified under illumination. It can be seen that the potential 1.15–1.20 V versus RHE can be selected as E_onset_, and the nanoflake film electrode behaves like a photocathode (p-type)^46,47^. It is well known that the negative shift of the onset voltage potential is suitable for cooperation with the anodes to construct a non-biased PEC water splitting cell^48^. The plot for j–V of the film is shown in Fig. 6b. This was obtained using linear sweep voltammetry. The ratio $\frac{{j}_{light}}{{j}_{dark}}$ for the film was approximately 33, and the photocurrent value of 1.9 mA cm^−2^ is amongst the highest values reported in the literature for CuO^47^.

Figure 6c depicts the Nyquist plot derived from the EIS test to study the transfer charge rate across the interface of the electrolyte and electrode. The diameter of the semicircle indicates the resistance for the charge transfer (R_ct_)^47,49^; hence, the smaller semicircle indicates a remarkable increase in electron conductivity. In the EIS of photoelectrodes, the diameter of semicircles presents the charge transfer or/and mass transfer resistance across the electrode/electrolyte interface^50^. The presence of one semicircle means the lack of faradic reactions, which means only charge transfer occurred across the Stern layer^51^. In another word, the bigger the semicircle, the more insulator the electrode. This enhanced charge transfer at the interface is associated with the photoinduced surge of carrier density^51^, proving the photocatalytic activity of the electrode in harvesting solar powder. The initial resistance value (known as the electrode resistance, or R_p_) for dark and illuminated conditions is identical at ~ 8 Ω. This value is related to the sum of the resistance of the working electrode and the contact resistance between the current collector and electrode^50^. The real resistance in the semicircle can be attributed to the mass transfer and charge transfer rates at the interface of the film and electrolyte^44^, where the value for the film under dark conditions (~ 11,000 Ω) is orders of magnitudes greater than that of under illumination (~ 90 Ω). This significant difference reflects the outstanding photocatalytic activity of the film, which gives it great potential for energy harvesting from sunlight.

According to Fig. 6a, the photoelectrode is not able to generate current beyond 1.15 V versus RHE, although 1.23 V is a thermodynamical requisite for splitting water. Hence, the performance of the photoelectrode film in the photocatalytic water-splitting process was measured by determining the employed bias photon to electron conversion effectiveness (ABPE) (where bias is varied between working and counter electrodes) using the following formula^52,53^:

7 $$ {\text{ABPE}}\;(\% ) = \left( {\frac{{\left[ {{\text{J}}_{{\text{p}}} \times \left( {1.23 - \left| {{\text{V}}_{{\text{b}}} } \right|} \right) \times\upeta _{{\text{F}}} } \right]}}{{{\text{P}}_{{{\text{total}}}} }}} \right) \times 100\% $$

where 1.23 V versus RHE indicates the minimum thermodynamic voltage for the splitting of water molecules^54^, *J*_*p*_ is the density of the photocurrent at the used bias voltage (mA cm^−2^), *V*_*b*_ is the applied bias voltage (V), *P*_*total*_ is the incident light's intensity (mW cm^−2^), and η_F_ is Faradic efficiency (taken in this study to be 0.8, with a conservative approach). Figure 6e represents the ABPE of the CuO nanoflake film. The measured efficiency peaks at 0.52 V with ABPE effectiveness of 1%, which is quite promising for water splitting. It should be noted that the water-splitting potential for a given material should not exceed the thermodynamic potential. No sacrificial donors or chemical bias were used in this analysis, and the bias of the electrodes counter along with the reference electrode was reported.

As a function of excitation wavelength, the incident photon-to-current efficiency (IPCE) corresponds to the ratio of the photocurrent and the rate of incident photons from a light source. Monochromatic light sources were employed to irradiate the electrode, and the IPCE factor was calculated using the following equation^45^:

8 $$ IPCE\;(\lambda ) = \left( {\frac{{\left| {J_{p} } \right| \times 1240}}{{P_{mono} \times \lambda }}} \right) $$

where 1240 nm is a multiplication of plank's constant (h), P_mono_ is the power intensity of the monochromated illumination (mW cm^−2^), and λ is the illuminated light's wavelength (nm).

The plot of IPCE versus wavelength is illustrated in Fig. 6d. IPCE of the film shows a continuous increase from 800 to 400 nm, which is more promising than the results in the literature^55,56^ for water-splitting applications. This increase implies that the bandgap of the nanoflakes is somewhat less than 1.6 eV, as the nanoflakes can be stimulated with the light of 800 nm wavelength. This bandgap could be due to the presence of NiO in the CuO phase and is in good agreement with previously derived results. The plot peaks at 432 nm to ~ 18% under − 0.3 V versus SCE. These values are greater than those reported in the literature^55,56^.

As two-electrode systems are employed in practical applications of photocatalytic materials, the j–V plot of the film electrode was plotted using a two-electrode system, where the Pt wire and film electrode were applied as counter and working electrodes, respectively. The results are plotted in Fig. 6f. Photocurrent flowed from − 0.3 V and peaked at ~ 1.2 V with a current density of − 1.9 mA cm^−2^, which is equal to that in the two-electrode system. The overall shape of the j–V plot for the two-electrode system implies that when external bias is applied, this nanomaterial can be effectively employed beyond the potential of − 0.3 V for water-splitting applications.

In this research, similar to the most studies presented in the Table S2, we indirectly measured the performance of the photoelectrode in water splitting via analysing the onset potential, comparing the dark current and under illumination photocurrent, percentage of ABPE in a potential window of 0.6 V, and percentage of IPCE between the wavelength of 400 to 800 nm (which is the light wavelength), and measuring the photocurrent at a two-electrode system, all shown in Fig. 6. These indirect methods are well-described in Ref.^54^.

It has been proved that the conduction band of nano CuO is not able to provide enough negative potential to generate hydrogen^57^. To decompose water molecules, the electrode performance needs to be consistent with the high-level conduction band side, which is normally found to be − 0.2 to − 0.6 V versus RHE in recent literature^57,58^. The energy band of NiO is positioned between the energy bands of CuO and Cu_2_O Y^58,59^. Consequently, adding a small amount of NiO can enhance the electrochemical performance of CuO. The photo-generated electrons are transferred to the copper oxide materials and trapped for hydrogen evolution, which further is improved by the liberation of NiO once there is a homogeneous distribution in the base materials. If there are any cathodic transient peaks in the photoelectron activity, the doping materials, such as NiO and Pt, can assist elimination of those unexpected transitions and make the process relatively steady, which upturns the efficiency of hydrogen evolution in water-splitting. To increase the significance, a comparative study of the results of this work with existing data in literature works is tabulated and presented in the supplementary (Table S2).

## Conclusion

Pure-phase CuO with in-situ NiO dopant was successfully synthesised by the thermochemical conversion and transformation of waste FPCBs. The CuO nanoflakes have been produced from the e-waste, FPCB, which has in situ NiO doping. The in-situ NiO doping occurred due to the presence of the Ni emulsion on the surface of the FPCB to protect this from the heat and environment. For the first time, the NiO doped CuO nanoflakes have been produced from the FPCB waste. The sources and the composition of this waste are consistent, which has been provided by a renowned FPCB manufacturer. These are the off cuts from the final production stage, where the large sheets of FPCBs are punched and cut to the desired shape and size, leaving behind a large amount of waste resources. To synthesise the NiO doped nanomaterials, previous researchers used pure NiCO_3_, Ni(NO_3_), and NiSO_4_, while in this research no extra raw materials are used for this purpose. The process is cost-effective as the Cu and Ni compounds are expensive and utilising the waste resources for these elements is beneficial.

A thermal disengagement technique at 500 °C was applied to recover pure Cu with a Ni surface finish. This was then transformed into NiO-doped CuO nanoflakes by chemical route. The synthesised nanoflakes have a refined 1D nanorod (~ 5 nm in width) structure, which combines to form nanoflakes (with width varying from ∼ 10 to 50 nm, and length from ∼ 30 to 80 nm). The CuO nanoflakes have a specific surface area of 115.703 m^2^ g^−1^, and the pore size distribution indicates that the CuO nanoflakes have a mesoporous structure with an average 6 nm pore diameter. The nanoflakes show about 70–75% reflectivity in the visible and infrared region with a bandgap less than 1.57 eV. The NiO-doped CuO photocathodes obtained photocurrents of 1.9 mA cm^−2^ at 0.05 V versus RHE when introduced to PEC testing under lighting. We expect that these findings will encourage further research on the application of materials derived from problematic e-waste to synthesise high-value nanomaterials with great industrial potential.

## Supplementary Information

Supplementary Information.

## Data Availability

Data to support the findings of this study is available from the corresponding author upon reasonable request.
