# Supplementary material for: Value-added fabrication of NiO-doped CuO nanoflakes from waste flexible printed circuit board for advanced photocatalytic application
Source: Sci Rep. 2022 Jul 16;12:12171. doi: 10.1038/s41598-022-16614-4 (PMC9288507; doi:10.1038/s41598-022-16614-4)
Supplement: Supplementary file 1 — Supplementary Information. [file 41598_2022_16614_MOESM1_ESM.docx]

Value-added fabrication of NiO-doped CuO nanoflakes from waste flexible printed circuit board for advanced photocatalytic application

*Rumana Hossain*, Rasoul Khayyam Nekouei, Abdullah Al Mahmood, and Veena Sahajwalla*

*Centre for Sustainable Materials Research and Technology*

*SMaRT@UNSW*

*School of Materials Science and Engineering,*

*UNSW Sydney, Australia*

Correspondence: *r.hossain@unsw.edu.au

**Supplementary:**


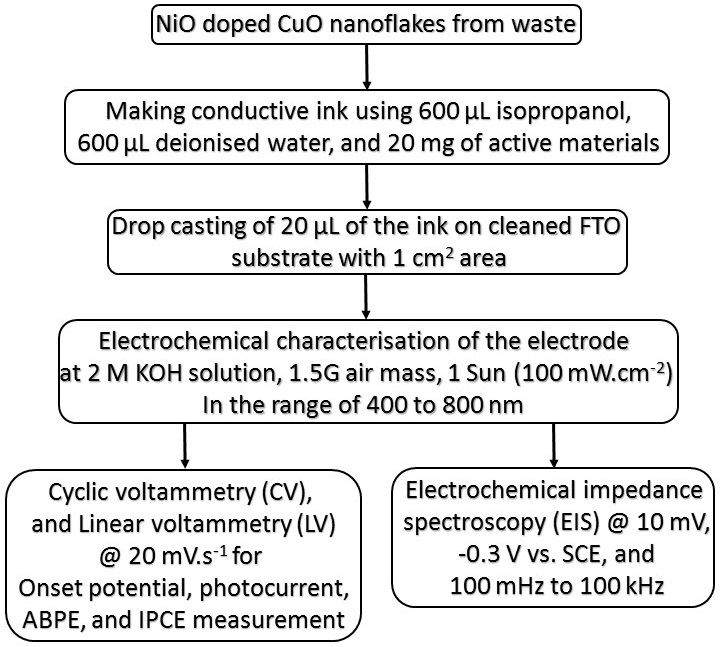


Figure S1: Scheme of electrode fabrication


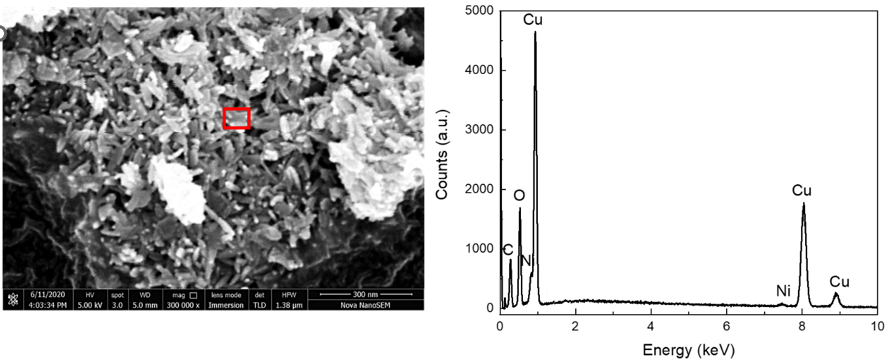


Figure S2. High resolution SEM images and EDX Spectroscopy of the selected area from CuO nanoflakes.


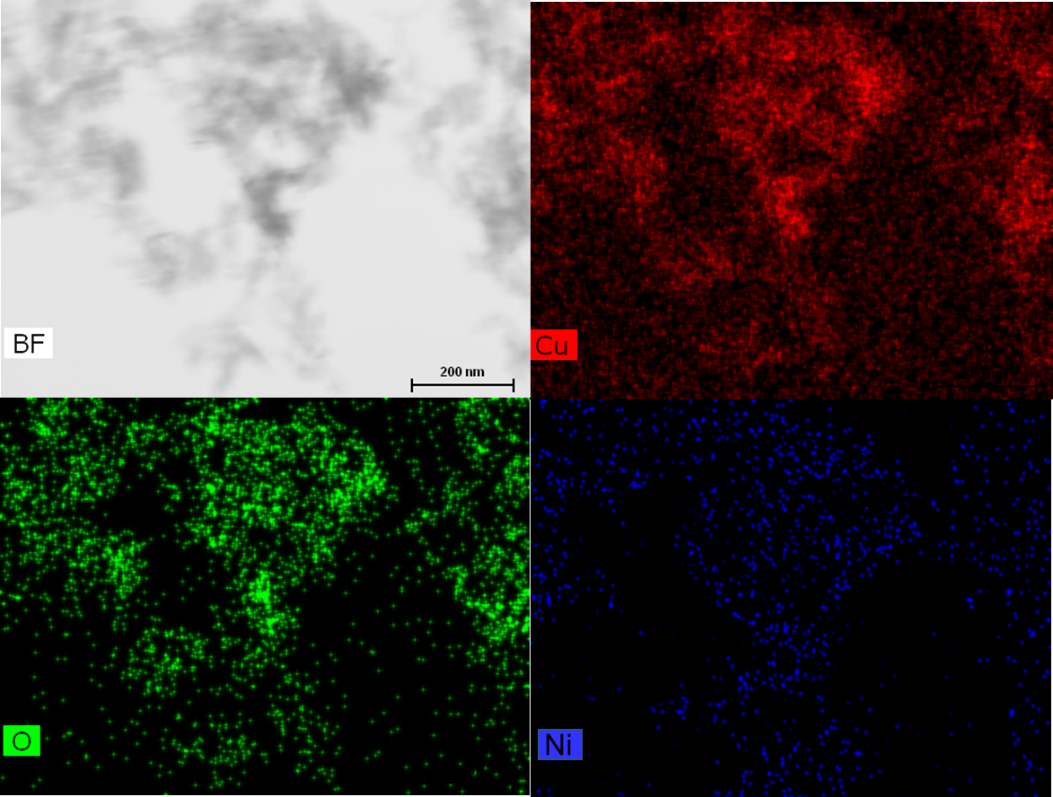


Figure S3. High resolution TEM images and EDX Spectroscopy of the selected area from CuO nanoflakes.


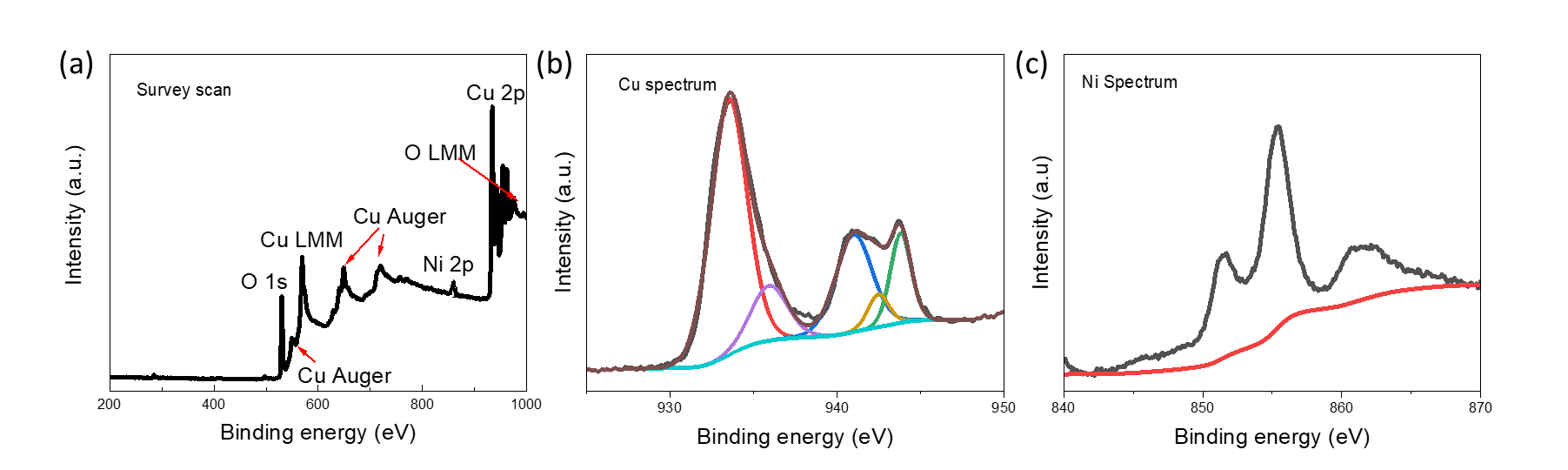


Figure S4: The XPS spectra of the nanoflakes. (a) Survey scan, (b) high resolution Cu spectrum, and (c) high resolution Ni Spectrum.

Table S1: Elemental analysis of the nanoflakes by ICP and XRF.

| ICP |  | XRF |  |
| --- | --- | --- | --- |
| Element | Wt% | Element | Wt% |
| Cu | 98.41 | Cu | 97.44 |
| Ni | 1.5 | Ni | 2.42 |
| P | 0.03 | P | 0.04 |
| Na | 0.01 | Na | 0.05 |
| S | 0.04 | S | 0.03 |
| Mg | 0.01 | Mg | 0.01 |

Table S2. Conditions and photocurrent of copper oxide photoelectrodes.

| Composition | Synthesis method | Substrate | Scan rate (mV.s^-1^) | Media and concentration | Photocurrent (mA.cm^-2^) | Ref. |
| --- | --- | --- | --- | --- | --- | --- |
| ZnO-CuO | Chemical process on Cu foil | Cu | - | Na_2_SO_4_, 100 mM | 0.5 @ -0.4 V vs. Ag/AgCl | [1] |
| p-CuO and p-CuO/n-CuWO_4_ | Electrochemical deposition followed by heat treatment | ITO | 10 | Na_2_SO_4_, 0.5 M | 0.6 @ -0.6 V vs. Ag/AgCl | [2] |
| CuO/g-C_3_N_4_ nanocomposite | Co-precipitation | Glassy carbon plate | - | - | 0.68 @ 1.2 V vs. Ag/AgCl | [3] |
| Cu_2_O/CuO/TiO_2_ | Electrochemical and dip coating deposition followed by heat treatment | Anodic Aluminum Oxide (AAO) template | 10 | Na_2_SO_4_, 1.0 M | 1.2 @ 0 V vs. RHE | [4] |
| CuO/TiO_2_/Pt | Electrochemical deposition | ITO | - | KOH, 1 M | 0.75 @ -0.5 V vs. Ag/AgCl | [5] |
| CuO | Sputtering followed by heat tretment | FTO | 50 | Na_2_SO_4_, 100 mM | 0.92 @ 0V vs. RHE | [6] |
| CuO | Electrochemical deposition followed by heat treatment | ITO | 5 | Na_2_SO_4_, 50 mM | 1.54 @ 0 V v. RHE | [7] |
| CuO | Hybrid microwave annealing | FTO | 10 | Na_2_SO_4_, 0.5 M | 4.4 @ 0 V vs. RHE | [8] |
| Cu_2_O/CuO | double-potential pulse chronoamperometric (r-DPPC) deposition | FTO | 10 | Na_2_SO_4_, 0.5 M | 3.15 @ 0.4 V vs. RHE | [9] |
| Cu/Cu_2_O/CuO | Chronoamperometric electrodeposition | FTO | 10 | KOH, 0.1 M | 2.6 @ -0.6 V vs. Ag/AgCl | [2] |
| Cu_2_O/CuO | Two-step electrodeposition followed by thermal annealing | ITO | - | Na_2_SO_4_, 1 M | 1.44 @ -0.7 V vs. Ag/AgCl | [10] |
| CuO | Thermal treatment | Cu | 30 | Na_2_SO_4_, 1 M | 1.4 @ 0 V vs. RHE | [11] |
| Cu_2_O/CuO | vacuum deposition system equipped with an electron-beam evaporation source | ITO | - | Na_2_SO_4_, 0.5 M | 0.24 @ -0.5 V vs. Ag/AgCl | [12] |
| CuO/Cu_2_O | Galvanostatic electrodeposition | FTO | 10 | Na_2_SO_4_, 0.5 M | 0.451 @ -0.3 V vs. Ag/AgCl | [13] |
| CuO | electrospinning | FTO | 5 | Na_2_SO_4_, 0.5 M | 0.16 @ 0.4 V vs. RHE | [14] |
| CuO/Cu_2_O | Electrochemical anodization followed by thermal tretemnt | Cu | 10 | Na_2_SO_4_, 0.5 M | 1.9 @ -0.3 V vs. Ag/AgCl | [15] |
| CuO | Sol-gel by manual dip coating | FTO | - | Na_2_SO_4_, 0.1 M | 1.52 @ 0 V vs. RHE | [16] |
| CuO | alkaline bath oxidation of Cu-foil | Cu | 10 | Na_2_SO_4_, 0.5 M | 1.3 @ 0 V vs. RHE | [17] |
| CuO | galvanostatic deposition | ITO | 5 | Na_2_SO_4_, 0.5 M | 0.55 @ -0.5 V Ag/AgCl | [18] |
| CuO | Sputtering | FTO | 50 | Na_2_SO_4_, 0.1 M | 0.92 @ 0 V vs. RHE | [6] |
| CuO | Spin coating | ITO | - | KOH, 1 M | 1.2 @ -0.55 V vs. Ag/AgCl | [19] |
| CuO nanoflakes with NiO doping | Thermo-Chemical Microrecycling | FTO | 20 | KOH, 2 M | 1.9 @ 0.05 V vs. RHE | This work |

References:

1. Wang, J., et al., *Hierarchically branched ZnO/CuO thin film with enhanced visible light photoelectrochemical property.* Materials Letters, 2015. 154: p. 44-46.

2. Zheng, J.Y., et al., *Facile preparation of p-CuO and p-CuO/n-CuWO 4 junction thin films and their photoelectrochemical properties.* Electrochimica Acta, 2012. 69: p. 340-344.

3. Ragupathi, V., et al., *CuO/g-C3N4 nanocomposite as promising photocatalyst for photoelectrochemical water splitting.* Optik, 2020. 208.

4. Huang, Q., et al., *Highly aligned Cu2O/CuO/TiO2 core/shell nanowire arrays as photocathodes for water photoelectrolysis.* Journal of Materials Chemistry A, 2013. 1(7): p. 2418-2425.

5. Xing, H., et al., *Exposing the photocorrosion mechanism and control strategies of a CuO photocathode.* Inorganic Chemistry Frontiers, 2019. 6(9): p. 2488-2499.

6. Masudy-Panah, S., et al., *Nanocrystal Engineering of Sputter-Grown CuO Photocathode for Visible-Light-Driven Electrochemical Water Splitting.* ACS Applied Materials and Interfaces, 2016. 8(2): p. 1206-1213.

7. Zhang, Z. and P. Wang, *Highly stable copper oxide composite as an effective photocathode for water splitting via a facile electrochemical synthesis strategy.* Journal of Materials Chemistry, 2012. 22(6): p. 2456-2464.

8. Jang, Y.J., et al., *Tree branch-shaped cupric oxide for highly effective photoelectrochemical water reduction.* Nanoscale, 2015. 7(17): p. 7624-7631.

9. Yang, Y., et al., *Cu2O/CuO bilayered composite as a high-efficiency photocathode for photoelectrochemical hydrogen evolution reaction.* Scientific Reports, 2016. 6.

10. Borkar, R., et al., *Copper Oxide Nanograss for Efficient and Stable Photoelectrochemical Hydrogen Production by Water Splitting.* Journal of Electronic Materials, 2018. 47(3): p. 1824-1831.

11. Li, J., et al., *Copper oxide nanowires for efficient photoelectrochemical water splitting.* Applied Catalysis B: Environmental, 2019. 240: p. 1-8.

12. Basnet, P. and Y. Zhao, *Tuning the CuxO nanorod composition for efficient visible light induced photocatalysis.* Catalysis Science and Technology, 2016. 6(7): p. 2228-2238.

13. Du, F., Q.Y. Chen, and Y.H. Wang, *Effect of annealing process on the heterostructure CuO/Cu2O as a highly efficient photocathode for photoelectrochemical water reduction.* Journal of Physics and Chemistry of Solids, 2017. 104: p. 139-144.

14. Einert, M., et al., *Electrospun CuO Nanofibers: Stable Nanostructures for Solar Water Splitting.* ChemPhotoChem, 2017. 1(7): p. 326-340.

15. John, S. and S.C. Roy, *CuO/Cu2O nanoflake/nanowire heterostructure photocathode with enhanced surface area for photoelectrochemical solar energy conversion.* Applied Surface Science, 2020. 509.

16. Kushwaha, A., et al., *Morphologically tailored CuO photocathode using aqueous solution technique for enhanced visible light driven water splitting.* Journal of Photochemistry and Photobiology A: Chemistry, 2017. 337: p. 54-61.

17. Ray, A., et al., *Optimization of photoelectrochemical performance in chemical bath deposited nanostructured CuO.* Journal of Alloys and Compounds, 2017. 695: p. 3655-3665.

18. Mahmood, A., F. Tezcan, and G. Kardaş, *Photoelectrochemical characteristics of CuO films with different electrodeposition time.* International Journal of Hydrogen Energy, 2017. 42(36): p. 23268-23275.

19. Chiang, C.Y., et al., *Copper oxide photocathodes prepared by a solution based process.* International Journal of Hydrogen Energy, 2012. 37(10): p. 8232-8239.
